# Performance evaluation of cefoxitin screen test on two different automated antimicrobial susceptibility test systems: a comparative study

**DOI:** 10.1128/spectrum.03815-23

**Published:** 2024-07-23

**Authors:** Ismail Yuceel-Timur, Cherilyn D. Garner, Simone Franklin, Dwight J. Hardy

**Affiliations:** 1 Global Medical Affairs, bioMérieux, Lyon, France; 2 Global Medical Affairs, bioMérieux, Hazelwood, Missouri, USA; 3 Department of Microbiology and Immunology, University of Rochester Medical Center, Rochester, New York, USA; University Paris-Saclay, Clamart, France

**Keywords:** VITEK 2, MRSA, *Staphylococcus *spp., cefoxitin screen, methicillin-resistant *Staphylococcus *spp., Phoenix, oxacillin, BD Phoenix, cefoxitin (oxsf02n)

## Abstract

Reliable detection of *mec*A and *mec*C-mediated beta-lactam resistance using automated antimicrobial susceptibility test systems is critical for patient care. The aim of this study was to compare the performance of the new cefoxitin screen test (oxsf02n) on the Vitek 2 card (Vitek 2) and BD Phoenix PMC-100 Gram-Positive AST Panel (Phoenix) against the reference method for the detection of *mec*A (and *mec*C)-mediated beta-lactam resistance. Two hundred fifty clinical fresh and stock *Staphylococcus* spp. isolates were included. There were 120 *mec*A-positive, 10 *mec*C-positive, and 120 *mec*A and *mec*C-negative isolates. Cefoxitin screen and oxacillin tests were performed on Vitek 2 and Phoenix and by their respective reference methods (disk diffusion for the cefoxitin screen test and broth microdilution for oxacillin) for all isolates. PCR testing was also performed to confirm the presence of *mec*A and/or *mec*C genes. Results from each system were compared to the reference methods. Statistical hypotheses were evaluated both for Vitek 2 compared to the reference methods and Vitek 2 compared to the Phoenix. Compared to the reference method, the Vitek 2 cefoxitin screen test had 100% sensitivity/98% specificity and the Phoenix cefoxitin screen test had 84% sensitivity/100% specificity for the detection of *mec*A (and *mec*C)-mediated beta-lactam resistance. When the oxacillin test was combined with the cefoxitin screen for Vitek 2, the sensitivity and specificity were unchanged. However, when the oxacillin and cefoxitin screen tests were combined for the Phoenix, the sensitivity increased to 100% and the specificity remained unchanged (100%). When considering cefoxitin alone, the Vitek 2 screen test showed a higher sensitivity than the Phoenix for the detection of *mec*A and *mec*C-mediated beta-lactam resistance. However, currently, both systems use a combination of the cefoxitin and oxacillin tests to interpret the final result, and both reached a high level of performance when cefoxitin and oxacillin results were combined.

**IMPORTANCE:**

This research marks the inaugural evaluation of the revamped cefoxitin screen test version in Vitek 2, juxtaposing it against reference methods and a primary competitor BD Phoenix.

## INTRODUCTION

*Staphylococcus* spp. are Gram-positive bacteria commonly found in humans and animals. *Staphylococcus* spp. strains can cause a range of infections, from minor skin infections to life-threatening diseases such as sepsis (1). One of the major challenges in treating infections caused by *Staphylococcus* spp. is the development of antibiotic resistance. Beta-lactam antibiotics are commonly used to treat staphylococcal infections. However, the emergence of *mec*A and *mec*C-mediated beta-lactam resistance has become a significant concern in the last 25 years (1). Methicillin-resistant *Staphylococcus aureus* (MRSA) is a well-known example of *mec*A-mediated beta-lactam resistance. The *mec*A gene encodes for the production of penicillin-binding protein 2a (PBP2a), which has a low affinity for beta-lactam antibiotics. As a result, these antibiotics are unable to bind to and inhibit the activity of PBP2a, leading to antibiotic resistance. MRSA strains are often resistant to 41 multiple antibiotics, making them difficult to treat (2). In recent years, *mec*C-mediated beta-lactam resistance has also emerged in *Staphylococcus* spp. The *mec*C gene, which encodes for a new variant of PBP2a, is primarily found in livestock-associated MRSA strains but has also been identified in human clinical isolates (3, 4). A combination of cefoxitin screen and oxacillin tests are commonly used in a clinical microbiology laboratory to detect the presence of *mec*A and/or *mec*C-mediated beta-lactam resistance. The cefoxitin screen test is widely used for the detection of methicillin resistance. It utilizes the susceptibility of the *Staphylococcus* spp. isolate to cefoxitin, which is a semi-synthetic cephalosporin antibiotic, as a surrogate to determine methicillin resistance. If the isolate is resistant to cefoxitin, then it is reported as methicillin-resistant and it is likely to have the *mec*A or *mec*C gene present, though there have been reports of other mechanisms of methicillin resistance related to changes in affinity for PBP (5). The oxacillin test is another test that is used to detect beta-lactam resistance in *Staphylococcus* spp. The test involves determining the susceptibility of the isolate to oxacillin, which is a penicillin-like antibiotic. If the isolate is resistant to oxacillin, it is reported as methicillin-resistant and may carry the *mec*A or *mec*C gene (6). However, it is important to note that the cefoxitin screen test and oxacillin test do not always provide accurate results when tested alone. As a result, the two tests are often tested in combination in clinical laboratories with the results being used together to determine methicillin resistance. Additionally, in some cases, confirmation of the presence of *mec*A or *mec*C genes may be necessary to eliminate a potential false negative or false positive result and detect correctly methicillin-resistant isolates (3), which would require additional testing, such as PCR-based assays (7). Furthermore, applying reference tests in routine is both time-consuming and labor-intensive, and laboratory technicians frequently face challenges due to insufficient training in utilizing these methods. Automated antimicrobial susceptibility testing systems are frequent options to test for the presence of *mec*A and *mec*C-mediated beta-lactam resistance. Vitek 2 and Phoenix are two examples of automated systems used for this purpose for *Staphylococcus* spp. (8). Both systems use the combination of the cefoxitin screen results and the oxacillin MIC together to determine the final interpretation reported for oxacillin. The redeveloped Vitek 2 cefoxitin screen test (oxsf02n) was approved for use by the FDA on 13 October 2022 and has been available in Japan since January 2024. The redeveloped version of cefoxitin has a lower time to result compared to the oxsf01n version and is more capable of detecting resistant isolates. The aim of this study was to evaluate the performance of the new cefoxitin screen test with and without combination of the oxacillin test for the Vitek 2 in comparison to the Phoenix for the detection of *mec*A and *mec*C-mediated beta-lactam resistance in *Staphylococcus* spp.

## MATERIALS AND METHODS

### Samples

From January 2020 to April 2022, a study was conducted that included 250 *Staphylococcus* spp. isolates, comprised of 148 *Staphylococcus aureus*, 65 *Staphylococcus epidermidis*, 10 *Staphylococcus haemolyticus*, 7 *Staphylococcus capitis*, 7 *Staphylococcus warneri*, 6 *Staphylococcus saprophyticus*, 5 *Staphylococcus lugdunensis*, and 2 *Staphylococcus simulans*. There were 120 *mec*A-positive, 10 *mec*C-positive, and 120 *mec*A and *mec*C-negative isolates, which were a combination of already characterized fresh (i.e., isolates collected up to 6 months after isolation from the clinical specimen) and stock (i.e., frozen isolates with no time constraints on the time from collection and they should be minimally subcultured) isolates according to the standard of care of the different institutions including Creighton University School of Medicine, International Health Management Associates, University of Rochester Medical Center, and University of California–Los Angeles. *mec*C-positive isolates were stock isolates of bioMérieux, and the other isolates were a mixture of contemporary and stock isolates. Previously characterized *mec*A and *mec*C-positive isolates were subjected to PCR testing for confirmation. Prior to antimicrobial susceptibility testing (AST), all 103 isolates were subcultured twice. Vitek 2 oxacillin and cefoxitin screen tests, cefoxitin disk diffusion (DD), and oxacillin broth microdilution were tested at the same time at varying sites during the clinical trial. Testing for the oxacillin and cefoxitin screen tests with the Phoenix was tested at a later time at URMC. Results from the Vitek 2 and Phoenix were compared to the reference method results (5). Any isolates with discrepancies between the results of Phoenix, Vitek 2, and the respective reference methods were repeated using both systems (Phoenix and Vitek 2) and reference methods starting from the same agar plate or initial suspension, if feasible. Both instruments used software to interpret cefoxitin and oxacillin results, and if one of the antimicrobial agents was resistant, the other was considered resistant as well. All isolates were subjected to tests with Vitek 2, Phoenix, reference method (disk diffusion or broth microdilution), and PCR test for *mec*A and *mec*C detection. Vitek 2 investigational use cards (with the oxacillin and cefoxitin screen oxsf02n test) and the BD AST Panel 449036 were used in the study, and CLSI breakpoints were applied to detect cefoxitin and oxacillin resistance. Statistical hypotheses were determined both according to the absence of statistically significant differences with a 95% confidence interval between the reference method and Vitek 2 in the alternative hypothesis and non-inferiority, which was the sensitivity and the specificity for both *mec*A and *mec*C using the Vitek 2 system, which is at least 95% of the sensitivity and specificity for the Phoenix system.

## RESULTS AND DISCUSSION

### Cefoxitin screen results

The Vitek 2 oxsf02n version of the cefoxitin screen test performance showed 100% sensitivity and specificity for *S. aureus*, *S. warneri*, *S. saprophyticus*, *S. lugdunensis*, *S. haemolyticus*, and *S. simulans*; 100% sensitivity and 83% specificity for *S. capitis*; and 100% sensitivity and 96% specificity for *S. epidermidis*. Phoenix cefoxitin screen test performance showed 100% sensitivity and specificity for *S. aureus*, *S. warneri*, *S. capitis*, *S. saprophyticus,* and *S. lugdunensis*; 71% sensitivity and 100% specificity for *S. epidermidis*; 89% sensitivity and 100% specificity for *S. haemolyticus;* and 0% sensitivity and 100% specificity for *S. simulans. mec*C-positive isolates were all *Staphylococcus aureus,* and the cefoxitin screen test had 100% sensitivity and specificity for Vitek 2 and Phoenix (S8 Supplementary Appendix).

### Oxacillin results

Vitek 2 oxacillin test performance showed 100% sensitivity and specificity for *Staphylococcus warneri*, *S. saprophyticus*, *S. lugdunensis*, *S. haemolyticus*, *S. capitis,* and *S. simulans*; 100% sensitivity and 96% specificity for *Staphylococcus epidermidis*; and 97% sensitivity and 100% specificity for *Staphylococcus aureus*.

Phoenix oxacillin test performance showed 100% sensitivity and specificity for *S. warneri*, *S. capitis*, *S. saprophyticus*, *S. lugdunensis*, *S. haemolyticus,* and *S. simulans* and 97% sensitivity and 100% specificity for *S. aureus* (S9 Supplementary Appendix).

### Combined results of cefoxitin and oxacillin

Vitek 2 showed 100% sensitivity and 98% specificity when applying the rule that if either cefoxitin or oxacillin is resistant, the final interpretation is that both antibiotics are resistant. However, there were errors in interpretation where one *Staphylococcus epidermidis* and one *Staphylococcus capitis* were incorrectly interpreted as resistant due to an oxacillin error, despite the sensitive result for oxacillin in the reference methods.

Phoenix demonstrated 100% sensitivity and specificity for both cefoxitin and oxacillin for all isolates when applying the rules.

### Applicability of AST systems to detect *mec*A (and *mec*C)-negative isolates

Analyzing resistance toward the cefoxitin screen test and oxacillin by AST systems, different susceptibility patterns and error types were observed according to the *mec*A and *mec*C status of the strains as shown in Table S1, S2 and S3.

Table S1 shows the results for the cefoxitin screen test and oxacillin together for *mec*A and *mec*C-negative isolates, as tested by Vitek 2, Phoenix, and reference methods. All isolates were negative for the cefoxitin screen test according to the reference method, and 3 out of 119 were resistant to oxacillin. There were 99% and 100% agreement with the reference method for both tests for the Vitek 2 and Phoenix methods, respectively. Two isolates were interpreted as resistant after forcing rules were applied in the Vitek 2 software while the results were susceptible by the reference methods.

### Applicability of AST systems to detect *mec*A-positive and *mec*C-negative isolates

In Table S2, for *mec*A-positive isolates, the highest agreement was observed for the Vitek 2 system, where all 120 isolates were correctly identified as resistant both with the cefoxitin screen test and oxacillin. The Phoenix system showed a lower percent agreement for the cefoxitin screen test with 84% of isolates correctly identified as resistant and 100% agreement for oxacillin.

### Applicability of AST systems to detect *mec*C-positive isolates

In Table S3, for *mec*C-positive isolates, both devices correctly identified all isolates as methicillin-resistant using the cefoxitin screen test, and 70% and 90% (Vitek 2 and Phoenix) of the isolates were correctly identified using oxacillin. The percentage agreement for these isolates was 100% for Vitek 2 and Phoenix when applying forcing rules.

Table S4 shows that the sensitivity for the cefoxitin screen test and oxacillin was 100% and 98%, respectively, for Vitek 2, and the specificity for the cefoxitin screen test and oxacillin was 98% and 99%, respectively. When combined, the sensitivity and specificity were at 100% and 98%, respectively. The overall accuracy for the cefoxitin screen test and oxacillin was 99% and 100% when combined. For the Phoenix system, there was high sensitivity for oxacillin (99%) and when combined (100%), but lower for the cefoxitin screen test alone at 84%. The specificity for the cefoxitin screen test and oxacillin together was 100%. The overall accuracy was at 90% for the cefoxitin screen test and 100% for oxacillin and 100% when the cefoxitin screen test and oxacillin were combined.

In this study, it was demonstrated that *mec*A and *mec*C-mediated resistance in *Staphylococcus* spp. could be effectively determined using a combination of the cefoxitin screen and oxacillin tests in automated AST systems. When cefoxitin and oxacillin were combined, both Vitek 2 and Phoenix instruments were efficient in detecting *mec*A and *mec*C-mediated resistance in *Staphylococcus* spp. using cefoxitin screen and oxacillin tests. However, discrepancies were observed between the devices and the reference methods, depending on the species and resistance profiles of the strains. However, the newly redeveloped Vitek 2 cefoxitin screen test exhibited higher sensitivity compared to Phoenix when used alone without using the oxacillin test.

The newly redeveloped Vitek 2 cefoxitin screen test exhibited 100% sensitivity, irrespective of the strains and resistant profiles (*mec*A and *mec*C-negative, *mec*A-positive, or *mec*C-positive), and a specificity of 98% with two cefoxitin screen-susceptible isolates being identified as resistant out of 250 isolates. In contrast, the Phoenix cefoxitin screen test performance showed 84% sensitivity across all 250 isolates. While no false positive results were found for the Phoenix cefoxitin screen test for *mec*A and *mec*C-negative isolates, repeat testing identified 20 *mec*A-positive cefoxitin screen-resistant isolates as susceptible (false negative). Among these, 18 isolates were *Staphylococcus epidermidis*; one was *S. simulans*, and the other was *S. haemolyticus*. These results indicate that the Phoenix cefoxitin screen test encounters difficulties in identifying *mec*A-positive isolates, particularly for *S. epidermidis* isolates. A study conducted by Mencacci et al. in 2009 shows that the cefoxitin disk diffusion method is less accurate than Phoenix in detecting *mec*A resistance in *S. epidermidis*, as 4 out of 11 methicillin-resistant isolates were wrongly classified as susceptible (8). Interestingly, our study yields opposite findings compared to this article. It should be noted that Phoenix demonstrated 100% sensitivity for the cefoxitin screen test in *mec*A and *mec*C-positive *S. aureus* isolates.

Better performance has been demonstrated by the newly redeveloped Vitek 2 cefoxitin screen in comparison to a previous study conducted in 2009. In the study by Junkins et al., the performance of the cefoxitin screen and oxacillin tests in both Vitek 2 and Phoenix for the detection of *mec*A-mediated resistance in *Staphylococcus aureus* was evaluated. Sensitivity and specificity values of 99.1% and 98.2% for the cefoxitin screen test and 100% and 99.4% for oxacillin, respectively, were reported (9). However, in our study, Vitek 2 exhibited 100% sensitivity and specificity in detecting *mec*A and *mec*C-mediated resistance in *S. aureus*. In another study by Bobenchik et al. in 2013, the performance of Vitek 2 in detecting resistance in *Staphylococcus* spp. was evaluated using the AST-GP71 card. In this study, the Vitek 2 cefoxitin screen test exhibited 100% sensitivity and specificity for *S. aureus* isolates and coagulase-negative *Staphylococcus* (10), which is consistent with our findings. However, it is important to note that this study utilized an older version of the cefoxitin screen test and included a limited number of strains for certain *Staphylococcus* spp. when compared to our study. Specifically, the numbers of strains included were as follows: *S. epidermidis* (*n* = 17) vs (*n* = 68), *S. haemolyticus* (*n* = 6) vs (*n* = 10), *S. capitis* (*n* = 4) vs (*n* = 10), and *S. warneri* (*n* = 1) vs (*n* = 7) (9). The current study has demonstrated the high performance of the Vitek 2 new cefoxitin screen test, especially when considering an increased number of rare strains.

Regarding the oxacillin results, Vitek 2 displayed 98% sensitivity, 99% specificity, and 99% accuracy, while Phoenix exhibited 99% sensitivity, 100% specificity, and 100% accuracy. Both instruments demonstrated efficient performance, with no significant difference between the results, i.e., non-inferiority (*P* < 0.05). Phoenix accurately characterized all *mec*A and *mec*C-negative isolates, whereas Vitek 2 had only one false positive result. Both instruments achieved 100% sensitivity and specificity in *mec*A-positive isolates. However, both instruments encountered some challenges with oxacillin tests in *mec*C-positive isolates, with Vitek 2 and Phoenix having 3 and 1 out of 10 isolates with false susceptible oxacillin results, respectively. However, these results are not surprising as false susceptible oxacillin results have been reported previously for *mec*C-positive isolates. More specifically, in a study conducted by Kriegeskorte et al., false susceptible oxacillin results were observed for 111 *mec*C-positive *S. aureus* isolates using both Vitek 2 and Phoenix systems. The most common pattern observed in the Phoenix and Vitek 2 systems was cefoxitin screen resistance and oxacillin susceptibility, with percentages of 54.1% and 83.8%, respectively (11). When considering the reference methods, all 111 isolates (100%) were correctly identified as *mec*C-positive when using cefoxitin DD; however, oxacillin broth microdilution showed that 43 (38.7%) of these isolates were susceptible, while 68 (61.3%) were resistant, demonstrating that even the reference method for oxacillin can struggle with identifying *mec*C-encoded resistance.

Although Vitek 2 had better sensitivity when using the cefoxitin screen test alone (100% Vitek 2 vs 84% Phoenix), overall, both systems performed acceptably for the detection of *mec*A and *mec*C-mediated resistance in *Staphylococcus* spp. when using both the cefoxitin screen and oxacillin tests combined, which is the standard of care in most laboratories.

### Limitations

The oxacillin version that is currently available for Vitek 2 (ox101n) and used in this study was developed through a comparison with the agar dilution reference method, and testing in this study for oxacillin and cefoxitin was done with different inocula for each instrument.

## AFTER EPUB

*[This article was published on 23 July 2024 with an error in the FDA approval date. The approval date was corrected in the first paragraph of the current version, posted on 8 August 2024.]*

## SUPPLEMENTAL MATERIAL

Tables S1-S9 spectrum.03815-23-s0001.docx Performance results before and after repeats.

ASM does not own the copyrights to Supplemental Material that may be linked to, or accessed through, an article. The authors have granted ASM a non-exclusive, world-wide license to publish the Supplemental Material files. Please contact the corresponding author directly for reuse.
