# Supplementary material for: Performance evaluation of cefoxitin screen test on two different automated antimicrobial susceptibility test systems: a comparative study
Source: Microbiol Spectr. 2024 Jul 23;12(9):e03815-23. doi: 10.1128/spectrum.03815-23 (PMC11370257; doi:10.1128/spectrum.03815-23)
Supplement: Tables S1-S9 — Performance results before and after repeats. [file spectrum.03815-23-s0001.docx]

Supplementary Appendix

Performance results after repeats

Table S1 Cefoxitin screen and oxacillin test results for 199 *mec*A (and *mec*C) negative isolates ^1,2^

**Cefoxitin screen/oxacillin** No. of isolates (% agreement) tested by^c^:

|  | **susceptibility pattern^a^** | VITEK® 2 | BD PHOENIX^TM^ | Reference (Ref) |
| --- | --- | --- | --- | --- |
|  | R/R | 1 (0.075) | 0 (0.0) | 0 (0.0) |
|  | R/S | 1 (0.075) | 0 (0.0) | 0 (0.0) |
|  | S/R | 3 (2.5) | 3 (2.5) | 3 (2.0) |
|  | Total R | 5 (4.2) | 3 (2.5) | 3 (2.0) |
|  | S/S | 114 (96.0) | 116 (98.0) | 116 (98.0) |
|  | Cefoxitin screen match  with Ref. | 118 (99) | 119 (100) | N/A |
|  | Oxacillin match with Ref. | 118 (99) | 119 (100) | N/A |

^a^R, resistant; S, susceptible.

*^b^*Positive agreement based on resistance to at least one of the compounds tested (cefoxitin screen or

oxacillin.)

*^c^*Results calculated following resolution of discrepancies after repeat testing.

Table S2 Cefoxitin screen and oxacillin test results for 120 mecA positive isolates^1^

|  |  |  |  |  |
| --- | --- | --- | --- | --- |
|  | Cefoxitin screen/oxacillin | No. of isolates (% | agreement) tested by^c^: |  |
|  | susceptibility pattern^a^ | VITEK 2 | Phoenix | Reference |
|  | R/R | 120 (100) | 100 (83) | 120 (100) |
|  | R/S | 0 (0.0) | 0 (0.0) | 0 (0.0) |
|  | S/R | 0 (0.0) | 20 (17) | 0 (0.0) |
|  | Total R^b^ | 120 (100) | 120 (100) | 120 (100) |
|  | S/S | 0 (0.0) | 0 (0.0) | 0 (0.0) |
|  | Cefoxitin screen match | 120 (100) | 100 (83) | N/A |
|  | with Ref. |  |  |  |
|  | Oxacillin match with Ref. | 120 (100) | 120 (100) | N/A |
|  |  |  |  |  |
|  | *^a^*R, resistant; S, susceptible. |  |  |  |

***^c^***Results calculated following resolution of discrepancies after repeat testing

^3^Please see S6 table in supplementary appendix for the initial result before repeat testing

**Table S3** Cefoxitin screen and oxacillin test results for *mec*C positive isolates^1^

Cefoxitin screen/oxacillin No. of isolates (% agreement) tested by^c^:

|  | susceptibility pattern^a^ | VITEK 2 | Phoenix | Reference |
| --- | --- | --- | --- | --- |
|  | R/R | 7(70) | 9 (90) | 10 (100) |
|  | R/S | 3 (30) | 1 (10) | 0 (0.0) |
|  | S/R | 0 (0.0) | 0 (0.0) | 0 (0.0) |
|  | Total R^b^ | 10 (100) | 10 (100) | 10 (100) |
|  | S/S | 0 (0.0) | 0 (0.0) | 0 (0.0) |
|  | Cefoxitin screen match  with Ref. | 10 (100) | 10 (100) | N/A |
|  | Oxacillin match with Ref. | 7 (70) | 9 (90) | N/A |

^a^R, resistant; S, susceptible.

^b^Positive agreement based on resistance to at least one of the compounds tested (cefoxitin screen or

oxacillin.)

^c^Results calculated following resolution of discrepancies after repeat testing

^1^Please see S7 table in supplementary appendix for the initial result before repeat testing

Table S4 Comparison of sensitivity, specificity and accuracy of VITEK 2 and PHOENIX systems for

cefoxitin screen and oxacillin test results compared to the reference method

|  |  | | | |
| --- | --- | --- | --- | --- |
|  | System and  agent tested | Sensitivity (%) | Specificity (%) | Accuracy (%) |
|  | VITEK 2 |  |  |  |
|  | Cefoxitin  screen | 100 | 98 | 99 |
|  | Oxacillin | 98 | 99 | 99 |
|  | Combined* | 100 | 98 | 100 |
|  | PHOENIX |  |  |  |
|  | Cefoxitin  screen | 84 | 100 | 90 |
|  | Oxacillin | 99 | 100 | 100 |
|  | Combined | 100 | 100 | 100 |
|  |  |  |  |  |

*Combination of oxacillin and cefoxitin screen test

Performance results before repeats

**TABLE S5** Cefoxitin screen and oxacillin test results for *mec*A (and *mec*C) negative isolates^1^

**Cefoxitin screen/oxacillin** No. of isolates (% agreement) tested by^c^:

| **susceptibility pattern^a^** | VITEK® 2 | BD PHOENIX^TM^ | Reference (Ref) |
| --- | --- | --- | --- |
| R/R | 1 (0.075) | 0 (0.0) | 0 (0.0) |
| R/S | 1 (0.075) | 0 (0.0) | 0 (0.0) |
| S/R | 3 (2.5) | 3 (2.5) | 5 (4.0) |
| Total R | 5 (4.2) | 3 (2.5) | 3 (2.0) |
| S/S | 114 (96.0) | 116 (98.0) | 114 (96.0) |
| Cefoxitin screen match  with Ref. | 118 (99) | 119 (100) | N/A |
| Oxacillin match with Ref. | 118 (99) | 119 (100) | N/A |

*^a^*R, resistant; S, susceptible.

*^b^*Positive agreement based on resistance to at least one of the compounds tested (cefoxitin screen or

oxacillin.)

***^c^***Results after repeats

**TABLE S6** Cefoxitin screen and oxacillin test results for *mec*A positive isolates

**Cefoxitin screen/oxacillin** No. of isolates (% agreement) tested by^c^:

| **susceptibility pattern^a^** | VITEK 2 | Phoenix | Reference |
| --- | --- | --- | --- |
| R/R | 120 (100) | 98 (81) | 119 (99) |
| R/S | 0 (0.0) | 1 (1) | 1 (1) |
| S/R | 0 (0.0) | 21 (18) | 0 (0.0) |

| Total R^b^ | 120 (100) | 120 (100) | 120 (100) |
| --- | --- | --- | --- |
| S/S | 0 (0.0) | 0 (0.0) | 0 (0.0) |

Cefoxitin screen match with Ref.

120 (100) 100 (83) N/A

Oxacillin match with Ref. 120 (100) 120 (100) N/A

|  | *^a^*R, resistant; S, susceptible. |
| --- | --- |
|  | *^b^*Positive agreement based on resistance to at least one of the compounds tested (cefoxitin screen or |
|  | oxacillin.) |
|  | ***^c^***Results calculated following resolution of discrepancies after repeat testing |
|  |  |
|  | **TABLE S7** Cefoxitin screen and oxacillin test results for *mec*C positive isolates |

**Cefoxitin screen/oxacillin** No. of isolates (% agreement) tested by^c^:

| **susceptibility pattern^a^** | VITEK 2 | PHOENIX | Reference |
| --- | --- | --- | --- |
| R/R | 6 (60) | 9 (90) | 10 (100) |
| R/S | 3 (30) | 1 (10) | 0 (0.0) |
| S/R | 0 (0.0) | 0 (0.0) | 0 (0.0) |
| Total R^b^ | 10 (100) | 10 (100) | 10 (100) |
| S/S | 0 (0.0) | 0 (0.0) | 0 (0.0) |
| Cefoxitin screen match  with Ref. | 10 (100) | 10 (100) | N/A |
| Oxacillin match with Ref. | 6 (0) | 9 (90) | N/A |
|  |  |  |  |

*^a^*R, resistant; S, susceptible.

*^b^*Positive agreement based on resistance to at least one of the compounds tested (cefoxitin screen or

oxacillin.)

^c^Results calculated following resolution of discrepancies after repeat testing

**S8. Cefoxitin Sensitivity and Specificity for *Staphlococcus spp*.**

| *Cefoxitin performance for*  *Staphylococcus spp*. | VITEK 2 | | PHOENIX | |
| --- | --- | --- | --- | --- |
|  | Sensitivity | Specificity | Sensitivity | Specificity |
| *S. aureus* | 100% | 100% | 100% | 100% |
| *S. epidermidis* | 100% | 96% | 71% | 100% |
| *S. warneri* | 100% | 100% | 100% | 100% |
| *S. capitis* | 100% | 83% | 100% | 100% |
| *S. saprophyticus* | 100% | 100% | 100% | 100% |
| *S. lugdunensis* | 100% | 100% | 100% | 100% |
| *S. haemolyticus* | 100% | 100% | 89% | 100% |
| *S. simulans* | 100% | 100% | 0% | 100% |

**S9. Oxacillin Sensitivity and Specificity for *Staphlococcus spp*.**

| *Oxacillin performance for Staphylococcus*  *spp*. | VITEK 2 | | PHOENIX | |
| --- | --- | --- | --- | --- |
|  | Sensitivity | Specificity | Sensitivity | Specificity |
| *S. aureus* | 97% | 100% | 97% | 100% |
| *S. epidermidis* | 100% | 96% | 100% | 96% |
| *S. warneri* | 100% | 100% | 100% | 100% |
| *S. capitis* | 100% | 100% | 100% | 100% |
| *S. saprophyticus* | 100% | 100% | 100% | 100% |
| *S. lugdunensis* | 100% | 100% | 100% | 100% |
| *S. haemolyticus* | 100% | 100% | 100% | 100% |
| *S. simulans* | 100% | 100% | 100% | 100% |
